# Ending a diagnostic odyssey—The first case of Takenouchi–Kosaki syndrome in an African patient

**DOI:** 10.1002/ccr3.3966

**Published:** 2021-03-02

**Authors:** Kaitlyn Flynn, Candice Feben, Lindiwe Lamola, Nadia Carstens, Amanda Krause, Zané Lombard, for DDD‐Africa as members of the H3Africa Consortium

**Affiliations:** ^1^ Division of Human Genetics, National Health Laboratory Service and School of Pathology, Faculty of Health Sciences University of the Witwatersrand Johannesburg South Africa

**Keywords:** *CDC42*, macrothrombocytopenia, Takenouchi–Kosaki syndrome, whole‐exome sequencing

## Abstract

First reported case of Takenouchi–Kosaki syndrome in an African patient with a *de novo* likely pathogenic missense variant identified in the *CDC42* gene.

## Graphical abstract

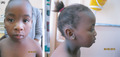

## 1 INTRODUCTION

Takenouchi–Kosaki syndrome (OMIM #616737), also known as Macrothrombocytopenia and Mental Retardation (MMR) syndrome, is an autosomal dominant, clinically heterogeneous disorder, characterized by multi‐systemic involvement, and developmental delay.^1^, ^2^ The syndrome first described in 2015, is associated with heterozygous pathogenic variants in the cell division control protein 42 homolog (*CDC42*) gene and has been described in a very limited number of individuals in the published literature.^1^, ^2^ The core phenotype includes intellectual disability, cardiac anomalies, sensorineural deafness, frequent infections, platelet abnormalities and structural brain defects; however, some affected individuals appear to present with a milder phenotype redolent of Noonan syndrome.^2^

## 2 CASE REPORT

**FIGURE 1:**
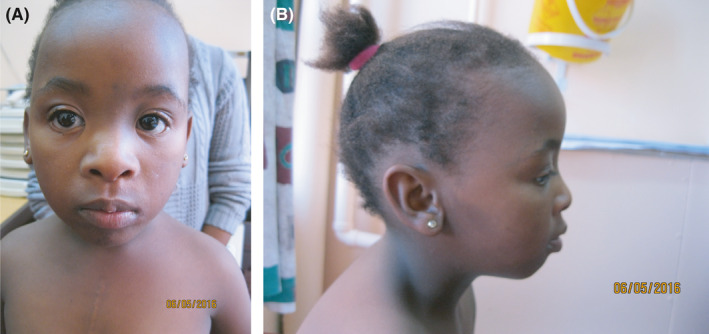
Facial features of proband. A, Frontal view. B, Side profile

The female proband was born to a nonconsanguineous couple with no medical complaints. The pregnancy was uncomplicated, apart from a threatened miscarriage at 16 weeks gestation, with no maternal illnesses or teratogenic exposures reported. The proband was delivered at term by Cesarean section, indicated for meconium‐stained liquor. Her birth weight was 2.9 kg (10th‐25th centile). Her other birth parameters were not available. She was admitted postnatally for two weeks with a complex congenital cardiac defect, shown on echocardiography to include transposition of the great arteries, an atrial septal defect, ventricular septal defect, and subpulmonic pulmonary stenosis. The infant was initially referred to the Genetics Clinic at the Rahima Moosa Mother and Child Hospital (RMMCH) in Johannesburg, South Africa, at the age of 6 months for an assessment, on the basis of her cardiac disease and associated dysmorphic features. At this assessment, she was noted to have failure to thrive, hypotonia and a number of unusual features, including a short neck with a thickened nuchal skin fold, a short nose with anteverted nares, low‐set posteriorly rotated ears, epicanthus inversus, a wide intermammillary distance and sparse scalp hair. She was assessed serially at the Genetic, Cardiac, Neurodevelopmental and Endocrine Clinics at the RMMCH over the next few years. At her most recent assessment, at 9 years, 8 months of age, she was microcephalic, with short stature and was noted to have dental malocclusion in addition to the previously documented dysmorphic features (Figure 1). Neurodevelopmental assessment identified inappropriate fine motor development, abnormal behavior (occasionally aggressive and hyperactive) and moderate intellectual disability. Baseline genetic testing included a CytoScan™ HD array (Thermo Fisher), which was reported as negative for any significant copy number variants. A computed tomography (CT) scan of the brain at 15 months of age showed no structural abnormalities and a renal ultrasound was normal. At 10 years of age, the patient was recruited to the Deciphering Developmental Disorders in Africa (DDD‐Africa) study for whole‐exome sequencing (WES). The DDD‐Africa study aims to evaluate exome sequencing in an African setting to allow for the sustainable integration of whole‐exome sequencing in developmental disorder diagnostics. The patient's family gave their informed consent prior to the patient's inclusion in the study. The research was approved by the Human Research Ethics Committee (Medical) of the University of the Witwatersrand (HREC: 190578).

**FIGURE 2:**
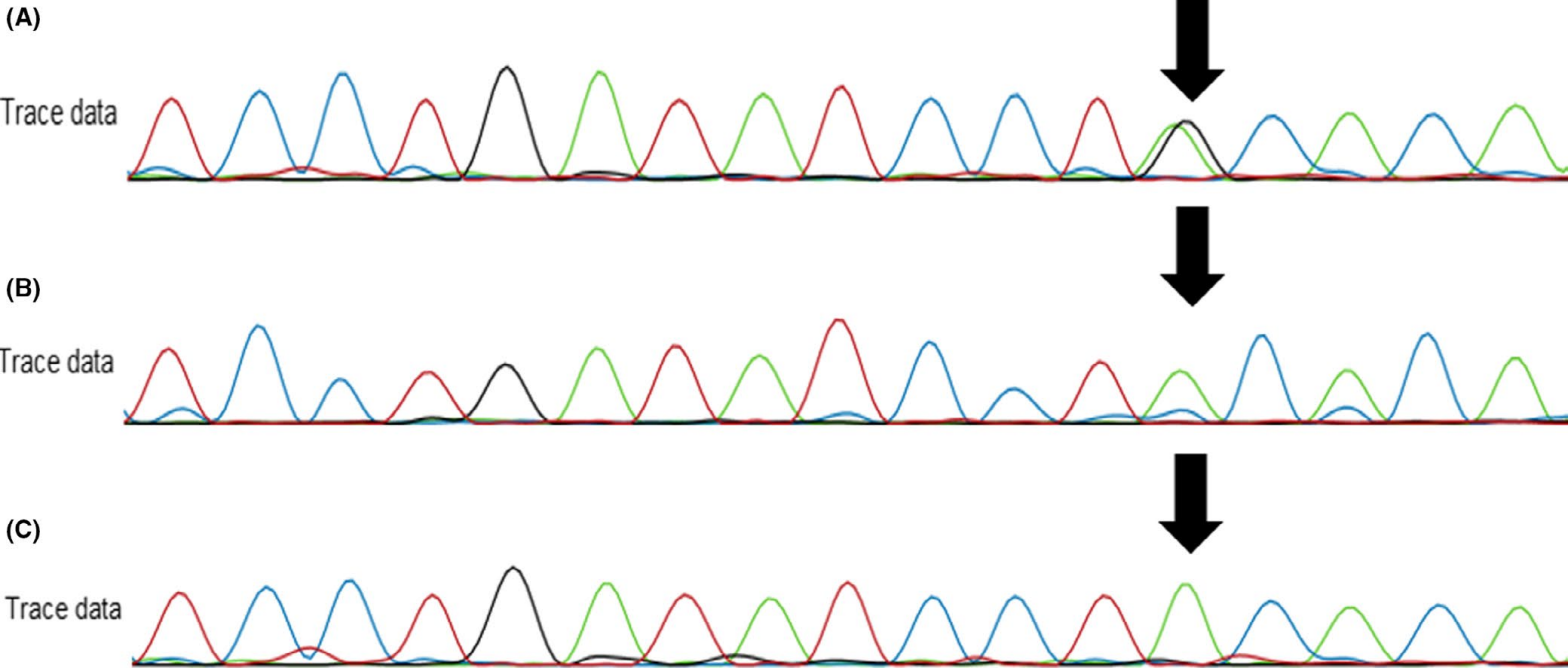
*CDC42* c.68A>G variant validation through Sanger sequencing. A, Electropherogram identifying the patient as an A/G heterozygote (variant is present). B, Electropherogram identifying the patient's mother as an A/A homozygote (variant is absent). C, Electropherogram identifying the patient's father as an A/A homozygote (variant is absent). The variant location is indicated by the black arrows

DNA was extracted from peripheral blood samples, using a modified version of the salting out method.^3^ WES was performed using the Thermo Fisher Ion Torrent S5™ next‐generation sequencing platform and the Ion Torrent AmpliSeq Exome RDY Kit (Thermo Fisher) as per the standard protocol. The average sequencing coverage of this sample was 150×. Data analysis was performed using the Moon (Invitae) software (v.3.2.0).^4^ A missense variant in the *CDC42* gene (NM_001791.4 c.68A>G, p.Tyr23Cys) was identified. The variant was validated through Sanger sequencing. Parental samples were sequenced and the data confirmed that both parents are homozygous for the reference allele indicating a *de novo* occurrence of the missense variant in the child (Figure 2).

Classification of the variant was performed following the recommendations and guidelines of the American College of Medical Genetics and Genomics and the Association for Molecular Pathology.^5^ Reviewed together with the clinical phenotype of this patient, we classified the p.Tyr23Cys variant as likely pathogenic (Class IV). The variant is located in the N‐terminal α‐Helix of the Ras domain^2^, ^6^ (PM1). The variant is absent in population databases^7^ (PM2) and has two pathogenic submissions in ClinVar^8^ (PP5; SCV000244190, SCV001250374). Available literature provides a well‐conducted in vivo, in vitro and in silico functional study that shows the variant to have a deleterious effect^2^ (PS3_Supporting). The *CDC42* gene has a low rate of benign missense variants and pathogenic missense variants are common^7^, ^9^ (PP2). In silico prediction tools suggest that the c.68A>G variant would have a deleterious effect on the protein^3^ (PP3). Sanger sequencing determined de novo inheritance of the variant, without maternity and paternity confirmed (PM6).

Based on the diagnosis of MMR syndrome in the proband, further recommendations for care were made, including an ophthalmologic assessment (for associated strabismus and optic atrophy), hearing test (for sensorineural hearing loss), full blood count and platelet count (to screen for thrombocytopaenia) and regular clinical spinal evaluation (for scoliosis).^2^

## 3 DISCUSSION

To our knowledge, this is the first reported case of MMR syndrome in an African patient. The phenotypic information presented contributes to the body of knowledge about this rare condition.

The *CDC42* gene encodes a small GTPase protein of the Rho‐subfamily, which is known to regulate signalling pathways, controlling multiple cellular functions such as migration, morphology, cell cycle progression, and endocytosis.^10^ The variant identified in this patient (c.68A>G) affects the 23rd amino acid (p.Tyr23Cys) of this 191 amino acid protein. The specific amino acid (Tyr23) is located at the N‐terminal alpha helix with the protein product present at the CDC42 surface and involved with PAK1 (P21 (RAC1) Activated Kinase 1) binding and WASP (Wiskott–Aldrich syndrome protein) binding to the GTPase.^2^, ^11^ The amino acid change (p.Tyr23Cys) results in reduced WASP interaction, as well as impaired binding to PAK1 and FMNL2 (formin like protein 2) as identified in the functional study conducted by Martinelli et al.^2^ The authors categorized known *CDC42* mutations into three groups according to their positions within the CDC42 structure. Based on the results reported in the same paper, the Tyr23Cys substitution is classified as a Group III *CDC42* mutation together with c.62T>C (p.Ile21Thr) and c.511G>A (p.Glu171Lys).^2^

A comparison of the clinical phenotype of a 14 year old patient with the p.Tyr23Cys variant^2^ and five patients with other Group III *CDC42* variants^2^ and our proband is presented in Table 1. Unlike reported patients with Group I and II variants, thrombocytopenia and dysmorphism resembling Noonan syndrome respectively, do not appear to be associated with Group III variants.^2^

**TABLE 1:** Comparison of clinical phenotype of reported patients with Group III pathogenic variants in *CDC42*

Clinical phenotype	Group III pathogenic variant^2^ N = 5	Tyr23Cys variant^2^	Proband
Growth abnormality			
Postnatal weight ≤ 2SD	0/3	+	+
Postnatal OFC ≤ 2SD	2/3 (66%)	+	+
Postnatal growth deficiency	3/5 (60%)	+	+
Intellectual disability	1/5 (20%)	Severe	Moderate
MRI brain abnormality	0/1	+	−
Seizures	0/5	+	−
Facial dysmorphism	5/5 (100%)	+	+
Cardiac anomalies	2/5 (40%)	−	+

## 4 CONCLUSION

Despite the identification of a complex cardiac abnormality at birth and the subsequent development of numerous clinical problems, including neurodevelopmental delay and poor growth, a diagnosis to explain the phenotype in our proband was only reached after a decade by using WES. Although there are numerous examples of the utility of WES in reaching diagnoses in individuals with multiple congenital anomalies and the use of WES as a first‐tier diagnostic test in these cases has been recommended,^12^ this testing modality remains unavailable to many individuals affected by genetic disorders in low‐ and middle‐income countries. The DDD‐Africa study is a research study aiming to fill this gap. The example of the use of WES technology in the proband presented here, one of the first patients in the study tested, provides convincing evidence of the need for this technology in under‐resourced countries to end the diagnostic odyssey faced by so many children with congenital anomalies. The implementation will additionally further fulfill the need to characterize developmental disorders in Africa, where they are largely undescribed and undocumented both clinically and molecularly.

## CONFLICT OF INTEREST

None declared.

## AUTHOR CONTRIBUTION

KF: conducted the literature search on the topic, performed the whole‐exome sequencing and variant validation, and drafted the initial version of the manuscript. CF: provided the clinical information, contributed to drafting and providing critical revision of the manuscript. LL, NC and AK: aided in clinical and variant interpretation and data analysis, and provided critical review of the manuscript. ZL: is the primary investigator of the DDD‐Africa study, conceptualized the study, raised the funds to perform the study, aided in variant interpretation and data analysis, and provided critical review of the manuscript.

## ETHICAL APPROVAL

Ethical approval was received from the Human Research Ethical Committee (Medical) at the University of the Witwatersrand, Johannesburg, South Africa for the DDD‐Africa study (M180678) as well as for the student substudy (M190578).

## Data Availability

The data that support the findings of this study are available on request from the corresponding author. The data are not publicly available due to privacy or ethical restrictions.
